# Oral Health-Related Quality of Life among Chinese Chronic Orofacial Pain Patients with Psychological Health Problems: A Moderated Mediation Model

**DOI:** 10.3390/ijerph20043244

**Published:** 2023-02-13

**Authors:** Ze-Yue Ou-Yang, Yao Feng, Yi-Fan Yang, Ning-Xin Chen, Xiao-Lin Su, Qian Zhang, Meng-Mei Zhong, Jing Hu, Qin Ye, Jie Zhao, Ya-Qiong Zhao, Yun Chen, Li Tan, Qiong Liu, Yun-Zhi Feng, Yue Guo

**Affiliations:** Department of Stomatology, The Second Xiangya Hospital, Central South University, Changsha 410011, China

**Keywords:** chronic orofacial pain, oral health-related quality of life, anxiety, depression, pain catastrophizing, moderate mediation model, oral health

## Abstract

Psychological therapies are important for comprehensive chronic orofacial pain (COFP) treatment. This study is to validate the effects of psychological factors on oral health-related quality of life (OHRQoL) among COFP patients in China. Pain catastrophizing, which is a subjective cognitive emotion used to manage the psychological aspects of pain among COFP patients, was examined in relation to COFP severity and OHRQoL. All 479 participants were recruited in Changsha, Hunan Province, China. Cronbach’s alpha coefficients (0.868–0.960), composite reliability scores (0.924–0.969), and average variance extracted from each construct (0.555–0.753) all indicated a good model fit. Pearson’s correlation analysis showed that age and education status have a positive correlation with COFP severity, pain catastrophizing, and anxiety. COFP severity was related to anxiety, depression, and COFP-OHRQoL. Pain catastrophizing was related to employment status. Anxiety and depression symptoms indirectly mediated the correlation between COFP severity and COFP-OHRQoL. As a second-stage moderator, pain catastrophizing moderated the mediating effects of anxiety symptoms and depression symptoms. Our findings suggest that anxiety, depression, and pain catastrophizing should be evaluated jointly to improve COFP-OHRQoL among COFP patients. This evidence will help therapists to comprehensively treat patients for the best treatment effect.

## 1. Introduction

The International Orofacial Pain Classification Committee proposed the International Classification of Orofacial Pain (ICOP) in 2020, which suggests that orofacial pain (OFP) disorders primarily involve the masticatory muscles, the temporomandibular joint, and associated structures of the head, face, and neck [1]. Approximately 20% of individuals in the United States suffer from OFP, and greater than 5% of these individuals can develop chronic orofacial pain (COFP) [2]. Chronic orofacial pain oral health-related quality of life (COFP-OHRQoL) is a measurement of how orofacial-related pain and discomfort affects physical, psychological, and social functions, along with well-being; previous studies have shown that patients with COFP typically experience months of suffering caused by pain [3,4]. COFP usually limits the movement of the jaw, which makes eating and speaking difficult [5]. Furthermore, reducing one’s ability to communicate has detrimental effects on an individual’s life because the mouth is also used as a language organ [6]. Additionally, because COFP patients suffer from long-term troubles, their work efficiency can be poor, and the cost of medical treatment can be high. These factors can directly or indirectly decrease COFP-OHRQoL [7]. Moreover, COFP does not respond well to current drug treatments because of the risk of side effects and addiction [8]. Strong scientific evidence shows that the treatment of COFP is highly effective when using noninvasive treatments such as physical self-regulation, psychosocial (cognitive and behavioral) self-regulation, and education [5]. In addition, the ICOP guidelines suggest that patients with COFP may adopt a biopsychosocial model, which describes key psychological and behavioral factors that may contribute to understanding current levels of pain and disability, and guides physicians so that cognitive behavioral therapy is enhanced, hence improving COFP-OHRQoL [1]. Studying the psychological factors of COFP patients is important to develop more effective treatments and management strategies and to lay the groundwork for future research in this area.

In recent years, psychological factors have received increasing empirical attention as key outcomes in the progression of COFP as well as COFP-OHRQoL. The importance of psychological factors is evident across a wide range of orofacial diagnoses [9], including musculoskeletal (e.g., temporomandibular disorders [10]), neurovascular (e.g., migraines [11]), and other types of OFP. Claudia M et al. proposed the influence of psychological factors on pain experience, pain expression, and pain inhibition, which result in pain-related disability [12]. Health-related quality of life (HRQoL) is declining as a consequence of both physical causes (such as pain) and mental health issues (such as anxiety and depression), and this is becoming increasingly evident as the incidence of COFP rises [13]. COFP can also cause anxiety or depression-like feelings, which, in turn, exaggerate the pain sensations of the patient [13]. Although there is a high prevalence of COFP in China [5], there are still no clinical studies in China that have examined the psychological factors associated with patients suffering from COFP. Therefore, it is crucial to investigate the potential processes and changeable psychological aspects linked with COFP development to aid clinicians in their understanding and management of COFP in a variety of settings.

Recent research has indicated that characteristics associated with anxiety and depression may have an additive influence on the prognosis for negative outcomes in individuals with COFP [13]. Clinical assessments show that those with chronic pain are three to five times more likely than the general population to suffer from anxiety [14]. Valeria Donisi et al. found that anxiety symptoms increased with COFP severity and were associated with lower levels of COFP-OHRQoL [15]. The relationship between depressive symptoms and COFP has also been studied in previous research [1]. COFP can induce depression [13], which not only severely impairs psychosocial functioning but also decreases the COFP-OHRQoL of COFP patients [16,17]. Although the pathway relationships between psychological factors in patients with OFP have now been partially explored, an important question that remains concerns how we can use our understanding of the relationships between these factors to develop, identify, and implement appropriate management strategies. Mediation analysis is a method of statistical analysis that examines the proposed causal mechanisms that explain relationships between variables and quantifies the effects of potential explanatory variables (e.g., anxiety and depression) on the relationship between an exposure (e.g., COFP severity) and an outcome (e.g., COFP-OHRQoL). Therefore, studies of anxiety and depression as mediators may provide novel directions for intervention techniques for COFP symptoms, providing both a theoretical basis and empirical support for the management and improvement of COFP-OHRQoL among COFP patients.

The concept of pain catastrophizing can be defined as a mental state that is based on the exaggeration of negative emotions resulting from a recent or anticipated pain experience [18,19]. Those who tend to exaggerate their discomfort are more likely to face a number of unpleasant side effects [20]. These symptoms heighten painful sensations and hinder sufferers’ ability to focus elsewhere. Pain catastrophizing, according to other research, is a distinct contributor to the subjective experience of pain intensity [21,22]. According to research by Sullivan MJ et al., pain catastrophizing is a powerful predictor of pain outcome and is linked to more intense pain [23]. John A. Sturgeon et al. found that changes in pain severity reliably predicted fluctuations in catastrophic pain, and the level of catastrophic pain can also be correlated with a patient’s OHRQoL [24]. Furthermore, some studies have indicated that negative affectivity and pain catastrophizing share a strong overlap. Anxiety and depression may be exacerbated by pain catastrophizing [25]. Pain catastrophizing has been shown to be the best predictor of anxiety and functional impairment among individuals with chronic pain, as shown by research by Tang et al. [26]. It is crucial to evaluate pain catastrophizing since it may play a significant role in the development and maintenance of COFP. It is also imperative to address pain catastrophizing when treating COFP. The moderated mediation analysis determines whether causal relationships between variables are dependent upon or interact with another variable [27]. Previously hypothesized patterns of moderation were incorporated into our study, resulting in moderated mediation, wherein a mediated effect is altered by a third factor [28]. Testing whether pain catastrophizing moderates the pathway relationship between anxiety and depression and COFP-OHRQoL among COFP patients will help simplify measurement and optimize clinical procedures.

Although previous studies have documented the positive associations between psychological health problems and COFP-OHRQoL among COFP patents, additional studies are needed regarding the psychological characteristics of Chinese COFP patients and the differences in influencing factors. Few studies have also examined how pain catastrophizing influences the connection between mental health issues and COFP patients’ COFP-OHRQoL. Additionally, this study is the first to examine pain catastrophizing, a subjective cognitive emotion, and to further refine its role in managing psychological factors among patients with COFP. This is important because it is the first study to validate the impact of psychological factors on COFP-OHRQoL in the COFP population in China, which should help clinicians better understand and manage COFP in a variety of settings.

**Figure 1:**
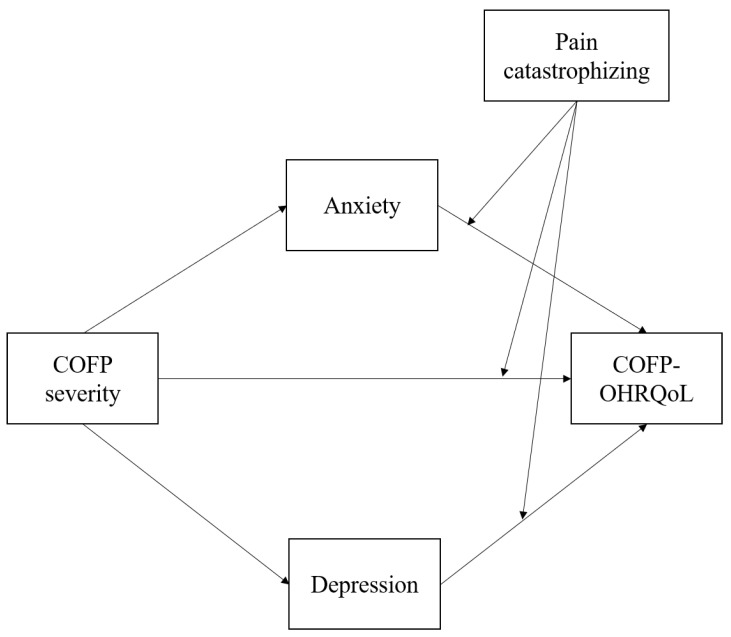
The theoretical moderated mediation model.

The primary goals of this study were to (1) investigate the associations between COFP severity, anxiety, depression, pain catastrophizing, and COFP-OHRQoL and (2) test a mediation model of the effect of COFP severity on COFP-OHRQoL, wherein the latter is influenced by the former (anxiety and depression) and is, in turn, moderated by the presence of pain catastrophizing. In accordance with previous research, three hypotheses were developed (Figure 1).

**Hypothesis 1.**  *COFP severity is positively related to COFP-OHRQoL.*

**Hypothesis 2.**  *Anxiety (Hypothesis 2a) and depression (Hypothesis 2b) mediate the effect of COFP severity on COFP-OHRQoL.*

**Hypothesis 3.**  *Pain catastrophizing moderates the direct and indirect relationships between COFP severity and COFP-OHRQoL via anxiety and depression. Specifically, pain catastrophizing buffers the mediating effect of anxiety on COFP-OHRQoL (Hypothesis 3a) and buffers the mediating influence of depression on the effect of COFP-OHRQoL (Hypothesis 3b).*

## 2. Materials and Methods

### 2.1. Participants

Subjects were enrolled in the study between April 2022 and September 2022 at the Second Xiangya Hospital of Central South University’s Stomatology Clinic. Subjects with COFP were diagnosed by two experienced stomatologists using the ICOP criteria [1]. The criteria for clinical referral were based on pain intensity and were set for the purpose of the research. Subjects were selected if they met the following criteria: they experienced at least one day of orofacial pain (OFP) in the two weeks before questionnaire’s completion and at least five days of OFP each month for at least three months. Subjects receiving anesthesia who also suffered from symptoms or diseases associated with altered pain perception (e.g., neurological or mental diseases, diabetes, or cardiovascular disease) or other disorders that might have impaired the research were not included. A total of 479 COFP subjects were ultimately included in this analysis. The Human Experiment and Ethics Committee of Second Xiangya Hospital, Central South University, authorized this investigation (KQ2019FY01). All contributors voluntarily provided written informed consent. Information that can be used to identify any individual subject was removed and replaced with generic data. Participants completed a survey meant to elicit data on demographics, pain catastrophizing, anxiety, depression, and COFP-OHRQoL. Table 1 displays the results of our demographic analysis in detail.

### 2.2. Measurement

#### 2.2.1. Covariates

Information about participants’ genders, ages, education status, and employment status were collected as covariates.

#### 2.2.2. COFP Severity

An individual’s current degree of pain, as well as the highest, lowest, and mean levels of pain felt during the preceding week, can be quantified using the Brief Pain Inventory (BPI) scale [29]. To obtain a sense of the relative degree of discomfort, we summed 4-item pain severity subscale and then divided that total by 4. The reported scores ranged from 0 to 10 based on the findings, with higher scores indicating more severe pain. The validity of the BPI scale in this group has been demonstrated by its extensive usage in research and clinical practice towards COFP patients [30]. Reliability was high for this scale in the present investigation, as shown by the Cronbach alpha coefficient of 0.868.

#### 2.2.3. Pain Catastrophizing Symptoms

When in pain, one may experience pain catastrophizing, a cognitive-emotional process characterized by excessive dwelling on the problem, exaggeration of the pain’s severity, and a sense of powerlessness [19]. Sullivan developed the Pain Catastrophizing Scale (PCS) in 1995 as part of the Coping Strategies Questionnaire, which is used to assess the degree of pain catastrophizing (ranging from 0 to 6, with higher scores indicating greater catastrophizing) [23]. This study adopted the Chinese version developed by the Chinese (Hong Kong) scholar Yap et al. in 2008 [31]. The scale contains a total of 13 items. Using a 5-point scale from 0 (absence at all) to 4 (frequently present), the total score is 52 points. The overall Cronbach’s alpha coefficient of the scale is 0.923.

#### 2.2.4. Anxiety Symptoms

The Generalized Anxiety Disorder Assessment (GAD-7) [32], a well-validated, seven-item questionnaire used for screening and diagnosing generalized anxiety disorder in clinical practice and research, was used to evaluate anxiety levels in this investigation. Responses on a four-point scale varied from “hardly ever” to “almost every day.” For the last two weeks, higher scores indicated higher levels of anxiety. Cronbach’s alpha coefficient for this scale in the present investigation was 0.916.

#### 2.2.5. Depressive Symptoms

As a primary care depression-screening tool, the Patient Health Questionnaire-9 (PHQ-9) is a self-report questionnaire [33]. Response options on the PHQ-9 were “never,” “many days,” “more than half the days,” and “almost every day.” Each item has a possible range of 0–27 (with scores ranging from 0–3). The PHQ-9 has diagnostic validity that is on par with that of tests performed in a clinical setting. Cronbach’s alpha coefficient for this scale in the present investigation was 0.918.

#### 2.2.6. COFP-OHRQoL

The impact of COFP on OHRQoL was assessed via a set of questions adapted from the Manchester Orofacial Pain Disability Scale (MOPDS) [34]. Subjects can give a score of 0 if pain does not occur at all, 1 if it occurs frequently, or if it occurs constantly (2 points). Increases in COFP-OHRQoL scores reflect a more severe degree of oral functional restriction and hence a poorer quality of life. COFP-OHRQoL has been evaluated using this scale in several sample populations [35]. Cronbach’s alpha coefficient was 0.960 in this investigation.

#### 2.2.7. Data Analysis

Hypothesis 1. COFP severity is positively related to COFP-OHRQoL.

Hypothesis 2. Anxiety (Hypothesis 2a) and depression (Hypothesis 2b) mediate the effect of COFP severity on COFP-OHRQoL.

Hypothesis 3. Pain catastrophizing moderates the direct and indirect relationships between COFP severity and COFP-OHRQoL via anxiety and depression. Specifically, pain catastrophizing buffers the direct effect of anxiety on COFP-OHRQoL (Hypothesis 3a) and buffers the mediating influence of depression on the effect of COFP-OHRQoL (Hypothesis 3b). IBM SPSS Statistics 21.0 (International Business Machines Corporation, Armonk, NY, USA), SPSSAU, and Mplus 8.3 (Mplus Beijing Tianyan Rongzhi Software Co., Ltd., Beijing, China) were used for all analyses [36]. Descriptive statistics were then computed across all variables. The second step was to determine the validity and reliability of the scales. Cronbach’s alpha coefficient and composite reliability (CR) values were used to investigate the reliability of the scale’s elements. Both a Cronbach alpha coefficient greater than 0.70 and a CR greater than 0.70 indicate high levels of composite reliability [37,38]. The AVE was calculated for each construct [39], and it was found to be greater than 0.5 in each case, indicating excellent convergent validity. This number needs to be larger than the correlation between the structures [37]. Third, we examined the pearson correlation coefficients between COFP severity, COFP-related COFP-OHRQoL, pain catastrophizing, anxiety, and depression to identify probable relevant factors for multivariate analysis. Both models used demographic information such as age, gender, education status, and employment status as control variables. Finally, we conducted analysis to examine the hypothesized connection between COFP severity, COFP-related COFP-OHRQoL, pain catastrophizing, anxiety, and depression. The association between COFP severity and COFP-related OHRQoL was modeled using a moderated mediation model, with depression and anxiety serving as mediators and pain catastrophizing acting as a moderator. The parameters for the model’s analysis were as follows: bootstrap = 10,000, estimator = ML, and type = GENERAL. 

## 3. Results

### 3.1. Demographic Characteristics

In this study, 479 subjects with COFP were included. Table 1 summarizes the information regarding the participants’ sociodemographic characteristics and BPI, PCS, GAD-7, PHQ-9, and MOPDS scores. Of the 479 participants, 57.62% were female. The average age of the subjects was 38.363 years old (Standard deviation = 16.536). Regarding the education status of the participants, 16.49% were unknown, 17.12% graduated with junior high school education, 12.53% graduated with high school education, and 53.86% graduated from college or higher. A total of 43.01% of the participants were employed. Among the participants, the overall means (Standard deviation) of the BPI-F, PCS, GAD-7, PHQ-9, and MOPDS scores were 3.085 (2.203), 16.914 (12.137), 7.879 (5.639), 10.071 (6.888), and 17.511 (13.017), respectively.

### 3.2. Examination of the Measurement Model and Structural Model

Scale components such as (a) construct reliability, (b) convergent validity, and (c) discriminant validity was examined. For this purpose, we utilized Cronbach’s alpha coefficient to determine how well the constructs were holding together internally and whether or not they were consistent with one another. Cronbach’s alpha coefficients in the current study varied from 0.868 to 0.960, indicating the good to acceptable reliability of the latent constructs (see Supplementary Table S1). The CR values were all greater than 0.70, indicating that the constructs were reliable. All AVE values were greater than the suggested value of 0.50 (see Supplementary Table S1), thus indicating high convergent validity. We compared the square root of the AVE to the absolute values of the correlation coefficients between each construct and the other constructs to assess discriminant validity. A square root of the AVE that is greater than the correlation coefficients between components is indicative of strong discriminant validity (see Supplementary Table S2).

#### Correlation of the Study Variables

Table 2 shows the pearson correlation analysis of the study variables. There were a number of significant correlations among the predictor, mediator, moderator, and outcome variables. Age and education status was positively correlated with COFP severity, pain catastrophizing, and anxiety. Employment status was positively correlated with COFP severity. Gender was not correlated with COFP severity, pain catastrophizing, depression, anxiety, or OHRQoL (see Table 2).

### 3.3. Mediating Effect of Anxiety and Depression on the Relationship between COFP Severity and COFP-OHRQoL

We developed a model of mediating effects for anxiety and depression based on our hypotheses. The direct effect of COFP severity on COFP-OHRQoL was 0.152 (95% confidence interval (CI): 0.056–0.248). Table 3 shows that COFP severity was positively correlated with anxiety (β = 0.590, *t* = 15.975, and *p* < 0.001), depression (β = 0.590, *t* = 15.975, and *p* 0.001), and COFP-OHRQOL (β = 0.152, *t* =3.105, and *p* < 0.05). Furthermore, COFP-OHRQoL was significantly associated with both anxiety and depression (β = 0.417, *t* = 9.660, and *p* < 0.001; β = 0.284, *t* = 6.957, and *p* < 0.001), indicating that depression and anxiety partially mediated the relationship between COFP severity and COFP-OHRQoL. Anxiety and depression accounted for 46.07 and 25.47, respectively, of all effects through mediation (Table 4). Therefore, both Hypotheses 1 and 2 were supported.

### 3.4. Moderating Effect of Pain Catastrophizing on the Mediating Effect

**Figure 2:**
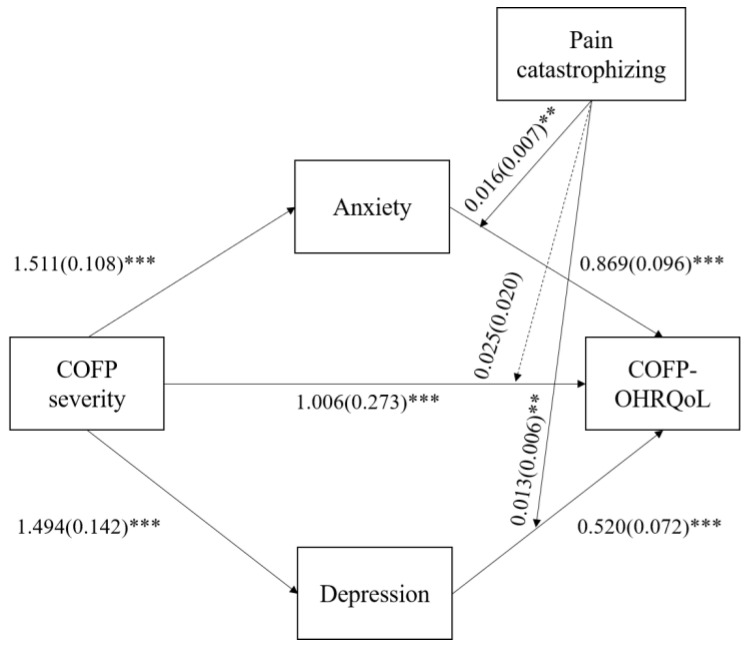
Diagram of the moderated mediation model. Note: Significant pathways are represented by solid arrows, and nonsignificant pathways are represented by dotted lines. Note: ** means *p* < 0.01; *** means *p* < 0.001.

According to Table 5, COFP severity has a positive effect on COFP-OHRQoL (β = 1.006, *t* = 3.678, and *p* < 0.001), although there is no evidence that COFP severity interacts with pain catastrophizing (β = 0.025, *t* = 1.215, and *p* = 0.224). COFP-OHRQoL and its interaction with pain catastrophizing were both significant predictors of anxiety (β = 0.869, *t* = 9.075, and *p* < 0.001; β = 0.016, *t* = 2.170, and *p* = 0.030) and depression (β = 0.520, *t* = 7.183, and *p* < 0.001; β = 0.013, *t* = 2.134, and *p* = 0.033). Figure 2 shows a visual representation of the moderated mediation model.

**Figure 3:**
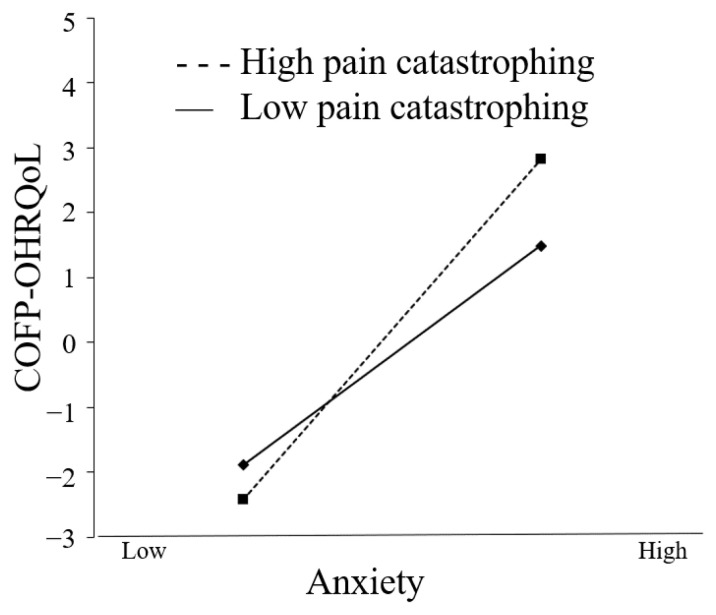
Moderating effect on the relationship between anxiety and COFP-OHRQoL.

**Figure 4:**
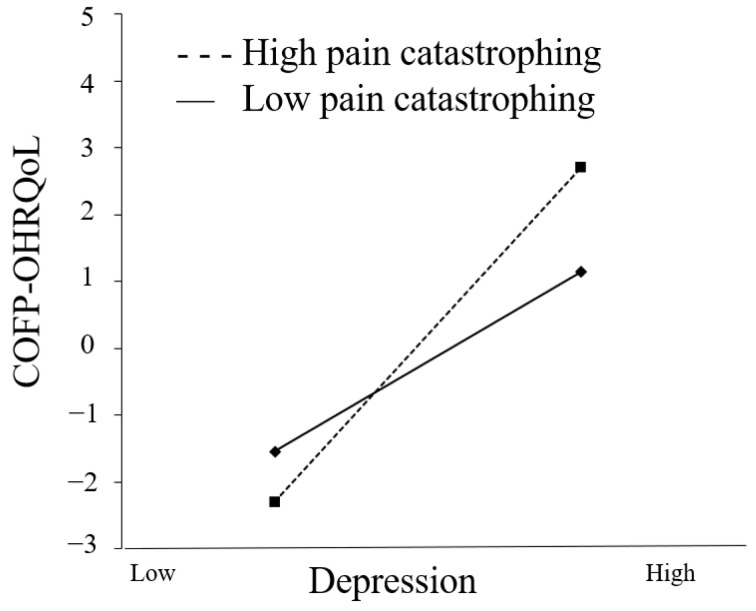
Moderating effect on the relationship between depression and COFP-OHRQoL.

Both Figure 3 and Figure 4 show how pain catastrophizing influences the correlation between depression and anxiety. The COFP-OHRQoL score improved significantly with increasing anxiety in both low and high pain-catastrophizing conditions (β = 1.018, *t* = 4.612, and *p* < 0.001; β = 1.609, *t* = 7.256, and *p* < 0.001), while depression increased in both low (β = 0.545, *t* =3.314, and *p* = 0.001) and high pain-catastrophizing conditions (β = 1.009, *t* = 5.332, and *p* < 0.001). There was a statistically significant difference in COFP-OHRQoL between the low and high pain-catastrophizing conditions (β = 0.591, *t* = 2.108, and *p* = 0.035); the COFP-OHRQoL score improved by 1.018 and 1.609 standard deviations for every one standard deviation increase in depression. The indirect effects were significantly different between those with low and high pain catastrophizing (β = 0.464, *t* = 2.043, *p* = 0.041), with anxiety increasing by 1 standard deviation and the COFP-OHRQoL score increasing by 0.545 (low pain catastrophizing) and 1.009 (high pain catastrophizing).

## 4. Discussion

This study used a large sample (*n* = 479) of Chinese COFP subjects to test the hypothesized relationships between COFP severity and COFP-OHRQoL as well as whether pain catastrophizing modified the direct and indirect associations between COFP severity and COFP-OHRQoL. This study not only addressed the gap in China regarding the internal mechanisms of COFP severity, pain catastrophizing, depression, and anxiety in relation to COFP-OHRQoL but also refined theories related to psychotherapy among COFP patients. Furthermore, this study is expected to provide Chinese clinicians with a comprehensive understanding and management of COFP patients across different circumstances.

The mean score of COFP severity was 3.085, which was lower than that of the BPI-F score of patients with facial pain syndromes reported by Lee, J. Y. et al. (5.03) [40]. Our results showed that the average pain catastrophizing score was 16.914, which indicates that participants with COFP had high levels of pain and generally experienced significant pain catastrophizing. In addition, previous studies have demonstrated a positive-to-moderate association between pain catastrophizing levels and pain severity among several types of COFP patients [41]. The average anxiety score of the participants was 7.879, indicating that the participants had anxiety. As in the case of Jonathan Greenberg’s research, COFP participants reported a high level of anxiety, with 56% reporting clinically significant anxiety symptoms [42]. The average depressive score among the study’s participants was 10.071. It is important to note that the data suggest an increased risk of anxiety and sadness among COFP-afflicted persons. Consistent with other studies, the participants’ average score on the COFP-OHRQoL indicated a low health-related quality of life, namely, 17.511 [43].

Additional social and other factors (covariates) may have affected our findings. In addition, we found evidence of a correlation between the potential mediation model variables. Thus, the Pearson rank correlation coefficient, a nonparametric measure of statistical dependence, was calculated. We found that age and education status were covariates; therefore, we controlled for these factors. We also used gender and employment status as covariates since we know they play a role in both research and practice [44,45].

This study developed a mediating model according to the hypothesis and the setting of the variables. The mediating model revealed that COFP severity was significantly positively associated with COFP-OHRQoL. Anxiety was positively correlated with COFP severity and COFP-OHRQoL. As a result, it is still crucial to accurately measure the levels of anxiety among patients in pain, as psychological variables have a major impact on the efficacy of pain management, especially for patients with chronic pain. Patients with greater anxiety in the treatment environment experience more pain; therefore, reducing anxiety during treatment is essential to reducing pain [46]. We found that COFP severity was positively associated with depression, which is in line with the study by Francesca Pistoia et al. [47] in which it was found that women with chronic migraines without a history of psychiatric comorbidities reported a higher tendency for depression. Additionally, depression had a negative influence on COFP-OHRQoL. It is suggested that screening and treatment for anxiety and depressive symptoms should be considered. Consultation with a multidisciplinary management service early on should be explored for these individuals to help in the treatment of these complicated afflictions.

Furthermore, we found that pain catastrophizing mediated the association between depression and COFP-OHRQoL and the relationship between anxiety and COFP-OHRQoL. Our results indicate that patients who experience high levels of pain catastrophizing experience persistent pain and reduced function at a higher rate than those who do not experience such symptoms. Pain catastrophizing has also been shown to forecast significant clinical symptoms such as more severe chronic pain severity and related disability [48]. The current study’s COFP respondents with high levels of depression and anxiety reported relatively high pain catastrophizing, which aligns with previous studies’ results [24]. Pain catastrophizing with respect to one’s suffering is one of the elements regularly linked to negative pain outcomes; in conjunction with depression, it may amplify the severity of pain in some painful situations [49]. It has also been shown that chronic pain patients whose symptoms are managed have better health outcomes when they minimize pain catastrophizing [50]. Thus, the early detection of psychosocial factors such as pain catastrophizing among this patient population is critical. Therefore, research on pain catastrophizing enriches the literature in relevant fields and provides novel therapeutic ideas.

One of the strengths of our study is its thorough identification of patients based on predefined criteria. We employed a number of indications linked to mental health issues, such as depression, anxiety, and exaggerated reactions to pain. This allowed us to more thoroughly assess the distinct links between different mental health issues and COFP. Furthermore, we found that the connection between COFP severity and COFP-OHRQoL was regulated by pain catastrophizing, which, in turn, was mediated by sadness and anxiety. High pain catastrophizers are more likely to suffer from depression and anxiety, which, in turn, has a greater negative effect on their COFP-OHRQoL than those in the low pain-catastrophizing group. Significant implications for the development of effective intervention techniques are suggested by these findings.

One limitation of this study is its cross-sectional design, which makes it impossible to draw any firm conclusions regarding the factors that could have contributed to the emergence of COFP.

This study has the potential to directly affect clinical practice by promoting an integrated treatment strategy that heavily relies on collaboration among a wide range of health providers, including psychologists. Evidence for the involvement of psychological and behavioral aspects in COFP clinical courses should be included in novel conceptual frameworks as part of a unified biopsychosocial approach to COFP management. It is possible that therapeutic cooperation may be strengthened by integrating patient education, behavioral therapies, and pharmaceuticals. This has the potential to alleviate COFP symptoms and decrease the need for medication.

## 5. Conclusions

A moderated mediation model was used in this investigation of the link between COFP severity and COFP-OHRQoL and its potential mediating psychological components. In summary, the results showed that depression and anxiety moderated the relationship between COFP severity and COFP-OHRQoL. High levels of pain catastrophizing forecasted COFP-OHRQoL according to the severity of depressive and anxious symptoms. The acquired results will allow Chinese doctors to better understand COFP and make better therapeutic decisions that benefit patients in an era of tailored COFP therapy.

## Figures and Tables

**Table 1:** Sociodemographic characteristics of Chinese COFP subjects.

Variable	Categories	*n* (%) or Mean ± Standard Deviation
Total	-	479 (100)
Age (years)		
	-	38.363 ± 16.536
Gender		
	Men	203 (42.38)
	Women	276 (57.62)
Education status, *n* (%)		
	Graduate degree and above	63 (13.15)
	Bachelor’s degree	195 (40.71)
	High school education	60 (12.53)
	Junior high school education	82 (17.12)
	Unknown	79 (16.49)
Employment status, *n* (%)		
	Studying	124 (25.89)
	Looking for a job	15 (3.13)
	Unemployed	134 (27.97)
	Employed	206 (43.01)
COFP Severity	-	3.085 ± 2.203
Pain catastrophizing	-	16.914 ± 12.137
Anxiety	-	7.879 ± 5.639
Depression	-	10.071 ± 6.888
COFP-OHRQoL	-	17.511 ± 13.017

**Table 2:** Pearson analysis between sociodemographic characteristics and COFP severity, pain catastrophizing, anxiety, depression, and OHRQoL.

Variables	1	2	3	4	5	6	7	8	9
1. Ages	1								
2. Genders	0.016	1							
3. Education status	0.523 **	−0.163 **	1						
4. Employment status	0.394 **	0.017	0.323 **	1					
5. COFP Severity	0.332 **	−0.009	0.342 **	0.141 **	1				
6. Pain catastrophizing	0.117 *	−0.041	0.221 **	0.113 *	0.124 **	1			
7. Anxiety	0.147 **	−0.004	0.209 **	0.075	0.582 **	0.250 **	1		
8. Depression	0.095 *	−0.025	0.119 **	0.025	0.454 **	0.152 **	0.383 **	1	
9. COFP OHRQoL	0.094 *	−0.004	0.125 **	0.030	0.503 **	0.334 **	0.603 **	0.506 **	1

Note: * *p* < 0.05, ** *p* < 0.01.

**Table 3:** Results regarding the mediation effect of anxiety and depression.

Outcome Variables	Predictors	R-Squared	*p*	β	Standard Error	*t*
Anxiety	COFP Severity	0.342	<0.001	0.590	0.037	15.975 ***
Depression	COFP Severity	0.210	<0.001	0.478	0.041	11.564 ***
COFP-OHRQoL	COFP Severity	0.465	<0.001	0.152	0.049	3.105 **
	Anxiety			0.417	0.043	9.660 ***
COFP-OHRQoL	COFP Severity			-	-	-
	Depression			0.284	0.041	6.957 ***

Note: ** *p* < 0.01, *** *p* < 0.001.

**Table 4:** The estimates of total, direct, and indirect effects of the model (with anxiety and depression as mediating variables).

Standardized Effect Size	Standardized Effect Size	Standard Error	95% Confidence Interval (CI)	Relative Effect Size
Total effects	0.534	0.040	0.452–0.607	
Direct effects	0.152	0.049	0.056–0.248	28.46%
Mediating effects				
Anxiety	0.246	0.030	0.191–0.309	46.07%
Depression	0.136	0.023	0.093–0.185	25.47%

**Table 5:** The moderating effect test of pain catastrophizing with respect to anxiety and depression.

Outcome Variables	Predictors	R-Squared	*p*	β	Standard Error	t	*p*	95% Confidence Interval
Anxiety	COFP Severity	0.342	<0.001	1.511	0.108	13.969	<0.001	1.285–1.712
Depression	COFP Severity	0.210	<0.001	1.494	0.142	10.504	<0.001	1.215–1.765
COFP-OHRQoL	COFP Severity	0.514	<0.001	1.006	0.273	3.678	<0.001	0.460–1.534
	Anxiety			0.869	0.096	9.075	<0.001	0.682–1.060
	Depression			0.520	0.072	7.183	<0.001	0.378–0.664
	Pain catastrophizing			0.210	0.037	5.663	<0.001	0.138–0.283
	COFP Severity × Pain catastrophizing			0.025	0.020	1.215	0.224	−0.015–0.064
	Anxiety × Pain catastrophizing			0.016	0.007	2.170	0.030	0.002–0.031
	Depression × Pain catastrophizing			0.013	0.006	2.134	0.033	0.001–0.024
Conditional mediating effect on anxiety at values of the moderator								
Low pain catastrophizing				1.018	0.221	4.612	<0.001	0.610–1.480
Mean pain catastrophizing				1.314	0.171	7.679	<0.001	1.004–1.674
High pain catastrophizing				1.609	0.222	7.256	<0.001	1.209–2.075
Differences between high and low pain catastrophizing				0.591	0.281	2.108	0.035	0.064–1.165
Conditional mediating effect on depression at values of the moderator								
Low pain catastrophizing				0.545	0.164	3.314	0.001	0.248–0.899
Mean pain catastrophizing				0.777	0.136	5.711	<0.001	0.533–1.067
High pain catastrophizing				1.009	0.189	5.332	<0.001	0.669–1.409
Differences between high and low pain catastrophizing				0.464	0.227	2.043	0.041	0.042–0.934

## Data Availability

The datasets generated during and/or analyzed during the current study are available from the corresponding author on reasonable request.

## Supplementary Materials

The following supporting information can be downloaded at https://www.mdpi.com/article/10.3390/ijerph20043244/s1: Table S1: Assessment of the measurement model; Table S2: Construct correlations and discriminant validity.
