# Supplementary material for: Oral Health-Related Quality of Life among Chinese Chronic Orofacial Pain Patients with Psychological Health Problems: A Moderated Mediation Model
Source: Int J Environ Res Public Health. 2023 Feb 13;20(4):3244. doi: 10.3390/ijerph20043244 (PMC9962568; doi:10.3390/ijerph20043244)
Supplement: Supplementary file 1 [file ijerph-20-03244-s001.zip › ijerph-2128795-supplementary.pdf]

Supplementary Table S1. Assessment of the measurement model.

Constructs/measurement items	Loading	Cronbach's α	CR	AVE
COFP severity（BPI）		0.868	0.924	0.753
BPI-1	0.876			
BPI-2	0.900			
BPI-3	0.859			
BPI-4	0.835			
Pain catastrophizing（PCS)		0.923	0.958	0.637
PCS-1	0.780			
PCS-2	0.811			
PCS-3	0.777			
PCS-4	0.817			
PCS-5	0.799			
PCS-6	0.815			
PCS-7	0.794			
PCS-8	0.758			
PCS-9	0.746			
PCS-10	0.823			
PCS-11	0.862			
PCS-12	0.829			
PCS-13	0.755			
Anxiety (GAD-7)		0.916	0.933	0.665
GAD-7(1)	0.772			
GAD-7(2)	0.854			
GAD-7(3)	0.757			
GAD-7(4)	0.821			
GAD-7(5)	0.788			
GAD-7(6)	0.819			
GAD-7(7)	0.888			
Depression (PHQ-9)		0.918	0.932	0.606
PHQ-9(1)	0.731			
PHQ-9(2)	0.817			
PHQ-9(3)	0.752			
PHQ-9(4)	0.793			
PHQ-9(5)	0.879			
PHQ-9(6)	0.778			
PHQ-9(7)	0.740			
PHQ-9(8)	0.777			
PHQ-9(9)	0.724			
COFP-OHRQoL (MOPDS)		0.960	0.969	0.555
MOPDS-1	0.763			
MOPDS-2	0.779			
MOPDS-3	0.694			
MOPDS-4	0.800			
MOPDS-5	0.747			
MOPDS-6	0.844			
MOPDS-7	0.845			
MOPDS-8	0.837			

MOPDS-9	0.825
MOPDS-10	0.762
MOPDS-11	0.727
MOPDS-12	0.687
MOPDS-13	0.719
MOPDS-14	0.749
MOPDS-15	0.744
MOPDS-16	0.754
MOPDS-17	0.724
MOPDS-18	0.703
MOPDS-19	0.673
MOPDS-20	0.716
MOPDS-21	0.708
MOPDS-22	0.740
MOPDS-23	0.747
MOPDS-24	0.581
MOPDS-25	0.702

Note: Cronbach's alpha (Cronbach'α);CR: composite reliability; AVE: average variance extracted.

Supplementary Table S2. Construct correlations and discriminant validity.

	COFP severity	Pain catastrophizing	Anxiety	Depression	OHRQoL
COFP severity	**0.819**				
Pain catastrophizing	0.124	**0.691**			
Anxiety	0.582	0.250	**0.782**		
Depression	0.454	0.152	0.383	**0.747**	
OHRQoL	0.503	0.334	0.603	0.506	**0.700**

Notes: The bold data represent the square root of average variance extracted (AVE).
